# RmtA, a Putative Arginine Methyltransferase, Regulates Secondary Metabolism and Development in *Aspergillus flavus*

**DOI:** 10.1371/journal.pone.0155575

**Published:** 2016-05-23

**Authors:** Timothy Satterlee, Jeffrey W. Cary, Ana M. Calvo

**Affiliations:** 1 Department of Biological Sciences, Northern Illinois University, Dekalb, IL, 60115, United States of America; 2 USDA, ARS, Southern Regional Research Center, New Orleans, LA, 70124, United States of America; Universidade de Sao Paulo, BRAZIL

## Abstract

*Aspergillus flavus* colonizes numerous oil seed crops such as corn, peanuts, treenuts and cotton worldwide, contaminating them with aflatoxin and other harmful potent toxins. In the phylogenetically related model fungus *Aspergillus nidulans*, the methyltransferase, RmtA, has been described to be involved in epigenetics regulation through histone modification. Epigenetics regulation affects a variety of cellular processes, including morphogenesis and secondary metabolism. Our study shows that deletion of *rmtA* in *A*. *flavus* results in hyperconidiating colonies, indicating that *rmtA* is a repressor of asexual development in this fungus. The increase in conidiation in the absence of *rmtA* coincides with greater expression of *brlA*, *abaA*, and *wetA* compared to that in the wild type. Additionally, the *rmtA* deletion mutant presents a drastic reduction or loss of sclerotial production, while forced expression of this gene increased the ability of this fungus to generate these resistant structures, revealing *rmtA* as a positive regulator of sclerotial formation. Importantly, *rmtA* is also required for the production of aflatoxin B_1_ in *A*. *flavus*, affecting the expression of *aflJ*. Furthermore, biosynthesis of additional metabolites is also controlled by *rmtA*, indicating a broad regulatory output in the control of secondary metabolism. This study also revealed that *rmtA* positively regulates the expression of the global regulatory gene *veA*, which could contribute to mediate the effects of *rmtA* on development and secondary metabolism in this relevant opportunistic plant pathogen.

## Introduction

The genus *Aspergillus* includes some of the most harmful fungal species known. *Aspergillus flavus* is one of these organisms, known mostly for its impact in agriculture. This fungus is an opportunistic pathogen of important oil seed crops, such as corn, peanuts, sorghum, cotton and treenuts. It efficiently disseminates in fields by producing asexual spores called conidia, infecting susceptible plants and contaminating them with highly carcinogenic mycotoxins, such as aflatoxins [1]. Once the fungus is established, formation of highly resistant structures termed sclerotia contribute to its survival under harsh environmental conditions [2]. Aflatoxins and other mycotoxins are thought to contaminate approximately one quarter of the world’s crops [3]. Economically, aflatoxin contamination leads to substantial losses each year mainly due to the necessary investments in the detection of infected crops and the removal of contaminated crops in developed countries. Both the U.S. Food and Drug Administration and the European Union have set limits on the amount of aflatoxins allowed in food and feed commodities of 20 parts per billion and 2 parts per billion respectively [4]. In developing countries lacking regulatory oversight for allowable levels of aflatoxin contamination in crops, adverse health impacts are also of concern due to ingestion of contaminated food or feed. This includes acute aflatoxicosis (aflatoxin poisoning) that can lead to jaundice, edema of the limbs, pain, vomiting, necrosis, and potentially acute liver failure or death [5–8]. Chronic aflatoxicosis can result in cancer (primarily liver cancer), immune suppression, stunted growth in children, and other pathological conditions [6, 8, 9]. Due to increasing climate change, outbreaks of aflatoxin contamination and those of other mycotoxins are predicted to become more prevalent worldwide [10]. Increased incidences of drought and higher temperatures can lead to conditions favoring *A*. *flavus* growth and aflatoxin production while weakening host plant defenses [11]. Furthermore, elevated carbon dioxide levels along with other environmental factors linked to climate change have been shown to cause increased expression of genes in the aflatoxin biosynthetic pathway [12].

Current approaches are insufficient to control crop colonization and aflatoxin contamination by *A*. *flavus*. It is possible that uncovering the regulatory mechanism governing aflatoxin production as well as those controlling *A*. *flavus* development and survival could provide novel genetic targets to be used in control strategies to decrease the detrimental effects caused by this fungus. Previous studies, particularly in *Aspergillus*, revealed that secondary metabolism, including the production of mycotoxins, and fungal development are genetically linked i.e. [13–17]. Examples of this coordinated regulatory mechanism are the putative methyltransferase LaeA and the global regulator VeA. These proteins, shown to interact with each other as part of a protein complex designated velvet [2, 18], are epigenetic regulators governing aflatoxin production, as well as conidiation and sclerotial formation in *A*. *flavus* [19–21]. Arginine methyltransferases (PRMTs) are epigenetic regulators that work through histone methylation. Arginine methylation of histones has been associated with transcriptional regulation, RNA processing and transport, signal transduction and DNA repair in mammals [22]. PRMTs transfer methyl groups from S-adenosylmethionine (SAM) to the guanidine nitrogen atoms of arginine [23]. This methylation results in a dimethylated arginine that can be in either an asymmetric or symmetric configuration [24]. These methylation patterns define two types of PRMTs: type I catalyze asymmetric dimethylation and type 2 catalyze symmetric dimethylation [24]. So far nine different PRMTs have been identified in humans, and homologs of three of these, PRMT1, PRMT3, and PRMT5, have been found to be conserved in other eukaryotes [24], including lower eukaryotes such as the yeast *Saccharomyces cerevisiae* [25] and the model filamentous fungus *Aspergillus nidulans* [26], where the genes encoding these proteins were designated *rmtA*, *rmtB* and *rmtC* [26]. Homologs of these PRMTs have not been previously characterized in *A*. *flavus*.

The present study focuses on elucidating the role of the PRMT1/*rmtA* homolog in *A*. *flavus*. Previous work showed that PRMT1/*rmtA* targets the amino-terminal tails of arginine 3 residue on the H4 histone inducing changes in chromatin structure in humans [27, 28] and in *A*. *nidulans* [23, 26] with a type I methylation pattern [26]. Several PRMT1/*rmtA* homologs have been further characterized in other fungi. In *S*. *cerevisiae*, mutations in Hmt1, the PRMT1/*rmtA* homolog, resulted in cold sensitive alleles [25]. Deletion of *rmtA* in *A*. *nidulans* causes growth reduction under conditions of oxidative stress [29]. In *Fusarium graminearum*, deletion of *amt1*, also a homolog of *rmtA*, lead to a reduction in vegetative growth, oxidative stress tolerance, virulence, and deoxynivalenol production [30]. *Neurospora crassa*, *amt-1* was necessary to sustain normal growth rates [31]. Our current studies in *A*. *flavus* revealed an indispensable role of *rmtA* in proper regulation of secondary metabolism, specifically aflatoxin biosynthesis, as well as in developmental processes, affecting conidiation and production of sclerotia in this agriculturally important fungus. It is possible that alteration of epigenetic regulation involving PRMTs could be used in novel control approaches to reduce the detrimental effects of *A*. *flavus* and of other fungal species.

## Materials and Methods

### Sequence Search, Alignment and Phylogenetic Analysis

The deduced amino acid sequence encoded by *A*. *flavus rmtA* (AFL2G_09078) was obtained from the Broad Institute Aspergillus Comparative Database. BLAST searches were performed on NCBI (http://www.ncbi.nlm.nih.gov/) using the blastp search tools to obtain the homologous sequences and corresponding e-values. The search was carried out using the *A*. *flavus* RmtA deduced protein sequence as query. To compare similarity and identity of RmtA to other homologs, a Needle pairwise sequence alignment was performed (http://www.ebi.ac.uk/Tools/psa/emboss_needle/). MUSCLE sequence alignment (http://www.ebi.ac.uk/Tools/msa/muscle/) was carried out with *A*. *flavus* RmtA as well as its homologs from other eukaryotes. This was followed by shading using the Boxshade tool version 3.21 (http://www.ch.embnet.org/software/BOX_form.html).

Phylogenetic analysis was performed with two groups of species. The first group included different fungal species: *Asperillus oryzae*, *Aspergillus terreus*, *Aspergillus fumigatus*, *Aspergillus kawachii*, *Aspergillus niger*, *Aspergillus nidulans*, *Neurospora crassa*, *Fusarium graminearum*, *Cyrptococcus neoformans*, *Schizosaccharomyces pombe*, *Saccharomyces cerevisiae*, *Candida albicans*, *Rhodosporidium toruloides*, *Puccinia graminis*, *Trichosporon asahii*, and *Coprinopsis cinerea*. The second group consisted of eukaryotic model organisms: *Schizosaccharomyces pombe*, *Saccharomyces cerevisiae*, *Aspergillus nidulans*, *Homo sapiens*, *Arabidopsis thaliana*, *Xenopus tropicalis*, *Danio rerio*, *Mus musculus*, *Drosophila melanogaster*, *and Caernorhabditis elegans*.

The software MEGA v6.0 was used for sequence alignment and analysis [32]. MUSCLE settings were used for multiple sequence alignment. For generation of phylogenetic trees, a Maximum-likelihood model was used with a bootstrap value of 1000 (http://megasoftware.net/).

### Strains and Growth Conditions

*Aspergillus flavus* CA14-wild-type (WT) (Δ*ku70*), CA14 (*pyrG*−, *niaD*−, Δ*ku70*) (SRRC collection # 1709), CA14-Δ*rmtA* (Δ*rmtA*::*pyrG*, Δ*ku70*), CA14-com-*rmtA* (Δ*rmtA*::*pyrG*, *rmtA*::*niaD*, Δ*ku70*), and CA14-OE*rmtA* (*gpdA(p)*::*rmtA*::*trpC(t)*::*pyrG*, Δku70) strains were used in this study. All strains were grown on Potato Dextrose Broth (PDB) medium at 30°C in the dark, unless specified differently. Agar (10 g/L) was added in the case of solid medium (PDA). Strains were maintained as 30% glycerol stocks at -80°C.

### Generation of the *rmtA* Deletion Strain (Δ*rmtA*)

An *rmtA* deletion cassette was generated by fusion PCR as previously described [33]. The 5’ and 3’ UTR fragments were first PCR amplified using primers Afl_rmtA_p1 and Afl_rmtA_p2, obtaining a 1.3 kb product corresponding to the 5’ UTR, and primers Afl_rmtA_p3 and Afl_rmtA_p4, obtaining a 1.6 kb fragment of the 3’UTR. For both reactions, *A*. *flavus* CA14 genomic DNA was used as template. These two DNA fragments were then PCR fused to another fragment corresponding to the *pyrG* marker from *Aspergillus fumigatus*. The intermediate fragment containing the marker was PCR amplified from plasmid p1439 using primers Afl_rmtA_p5 and Afl_rmtA_p6. Primer pair AFL_RMTA_F and AFL_RMTA_R was used for the final fusion PCR step. All the primers used in this study are listed in S1 Table. Fungal transformation was performed using *A*. *flavus* CA14 as the host strain as previous described [34]. Transformants were selected on Czapek Dox (CZ, Difco, Franklin Lakes, New Jersey, USA) plus sucrose as osmotic stabilizer without supplementation of uridine and uracil. Transformants were confirmed by Southern blot analysis. A selected deletion *rmtA* strain was then transformed with plasmid pSDS2.2, containing *niaD* ^*A*. *fumigatus*^ to obtain a prototroph.

### Generation of the *rmtA* Complementation Strain

A complementation strain was obtained by transforming the *A*. *flavus* Δ*rmtA* mutant with the *rmtA* wild-type allele. The complementation vector was generated as follows: a DNA fragment contained the entire *rmtA* coding region and 5′ and 3′ UTRs was first PCR amplified with primers comp RMTA_flavus_F and comp RMTA_flavus_R (S1 Table) from CA14 genomic DNA. Then, the PCR product was digested with NotI and PstI and ligated to the pSD52.2 vector, previously digested with the same restriction enzymes. pSD52.2 contains the *niaD*^*A*. *fumigatus*^ transformation marker. The resulting plasmid was designated pSD52.2-*rmtA*-com. This vector was then transformed into Δ*rmtA*, and the transformants were selected on CZ medium using nitrate as the sole nitrogen source. Complementation was confirmed by diagnostic PCR using OE_RMTA_F and OE_RMTA_R (S1 Table).

### Generation of the *rmtA* Over-Expression Strain (OE*rmtA*)

To generate the *rmtA* over-expression strain, the entire *rmtA* coding region was PCR amplified from CA14 genome using the OE_RMTA_F and OE_RMTA_R primers (S1 Table). The PCR product was then digested with AscI and NotI and ligated into the previously digested pTDS.1 plasmid, containing an *A*. *nidulans gpdA* promoter and *trpC* terminator, as well as the *pyrG*^*A*. *fumigatus*^ marker. This resulted in the generation of plasmid pTDS.1rmtAOE. The vector was then transformed into CA14. Confirmation of plasmid integration in the transformants was performed by diagnostic PCR using primers OE_RMTA_F and OE_RMTA_R. A selected transformant was converted into a prototroph by transforming the strain with pSDS2.2.

### Morphological Studies

*Aspergillus flavus* wild type, Δ*rmtA*, complementation and OE*rmtA* strains were point-inoculated on 30 ml of PDA medium and incubated in the dark at 30°C. Fungal growth was measured as colony diameter (mm) each day. To quantify conidial and sclerotial production, 10^6^ spores/ml were inoculated into 5 ml of melted PDA top agar and placed onto a 25 ml PDA solid medium. Cores (7 mm diameter) were collected to quantify conidia after 48 h and 72 h and 7 days, homogenized in water and counted using a hemocytometer (Hausser Scientific, Horsham, PA) under a Nikon Eclipse E-400 bright-field microscope (Nikon Inc., Melville, NY, USA). Sclerotial cores (16 mm diameter) were collected 24 days after inoculation, washed with 70% ethanol to eliminate conidiophores and counted using a Lieder stereo-zoom microscope. Experiments were performed in triplicate.

For sclerotial morphological analysis, strains were point-inoculated on 35 ml of PDA, on GMM [35] with 2% sorbitol, and on Wickerham medium (2 g yeast extract, 3 g peptone, 5 g corn steep solids, 2 g dextrose, 30 g sucrose, 2 g NaNO_3_, 1 g K_2_HPO_4_∙3H_2_O, 0.5 g MgSO_4∙_7H_2_O, 0.2 g KCl, 0.1 g FeSO_4∙_7H_2_O, 15 g agar per liter—pH 5.5) [36]. The strains were incubated at 30°C. Micrographs were obtained using a Leica MZ75 dissecting microscope attached to a Leica DC50LP camera (Leica Microsystems Inc., Buffalo Grove, IL, USA). Micrographs were taken from the cultures after an ethanol (70%) wash to remove conidiophores. The experiment was also performed with 3 replicates.

### Aflatoxin Analysis

Aflatoxin was extracted from top-agar inoculated cultures (three cores—16 mm diameter) with 5 ml chloroform. The chloroform phase was then collected and allowed to evaporate overnight. Dried residues were resuspended in 300 μl of chloroform. Twenty-five microliters of each extracts were separated by thin layer chromatography (TLC) as previously described [37] on silica plate (Si250F, J.T. Baker) using chloroform:acetone (85:15,v/v) as solvent system. Then plates were allowed to air dry, sprayed with 12.5% AlCl_3_ solution in 95% ethanol and baked at 80°C for 10 minutes. Presence of aflatoxin was detected under ultraviolet light at a wavelength of 375 nm. The aflatoxin B_1_ standard was purchased from Sigma-Aldrich (Sigma-Aldrich, St. Louis, MO, USA).

### Oxidative Stress Tolerance

PDA medium plates containing different concentrations of menadione (0 mM, 5 mM, 7.5 mM, 10 mM, 12.5 mM, and 15 mM) were point-inoculated with the *A*. *flavus* wild type, Δ*rmtA*, complementation and OE *rmtA* strains. Cultures were incubated for 48 h in the dark at 30°C.

### Gene Expression Analysis

Petri dishes containing 25 ml of PDB (Potato Dextrose Broth) were inoculated with conidia (10^6^ spores/ml) of *A*. *flavus* wild type, Δ*rmtA*, complementation and OE*rmtA* strains. Cultures were incubated in liquid stationary conditions at 30°C in the dark. Total RNA was extracted from lyophilized mycelial samples using TRIsure (Bioline, Taunton, MA, USA) reagent according to the manufacturer instructions. Gene expression analysis was performed either by Northern blot or qRT-PCR. For Northern blots, probe templates of *aflM/ver-1* were amplified by PCR from *A*. *flavus* genomic DNA using primers ver1-Nor-S and ver1-Nor-A and labeled with dCTP^p32^ (S1 Table). For qRT-PCR, five micrograms of total RNA was treated with RQ1 RNase-Free DNase (Promega. Madison, WI, USA). cDNA was synthesized with Moloney murine leukemia virus (MMLV) reverse transcriptase (Promega, Madison, WI, USA). qRT-PCR was performed with the Applied Biosystems 7000 Real-Time PCR System using SYBR green dye for fluorescence detection. cDNA was normalized to *A*. *flavus* 18S ribosomal gene expression, and the relative expression levels were calculated using the 2^-ΔΔCT^ method [38]. Primer pairs used are indicated in S1 Table.

### Statistical Analysis

Statistical analysis was applied to analyze all of the quantitative data in this study utilizing ANOVA (analysis of variance), in conjunction with a Tukey's multiple comparison testusing a *p*-value of *p* < 0.05 for samples that are determine to be significantly different.

## Results

### RmtA Is Conserved in Other Eukaryotes

**Fig 1:**
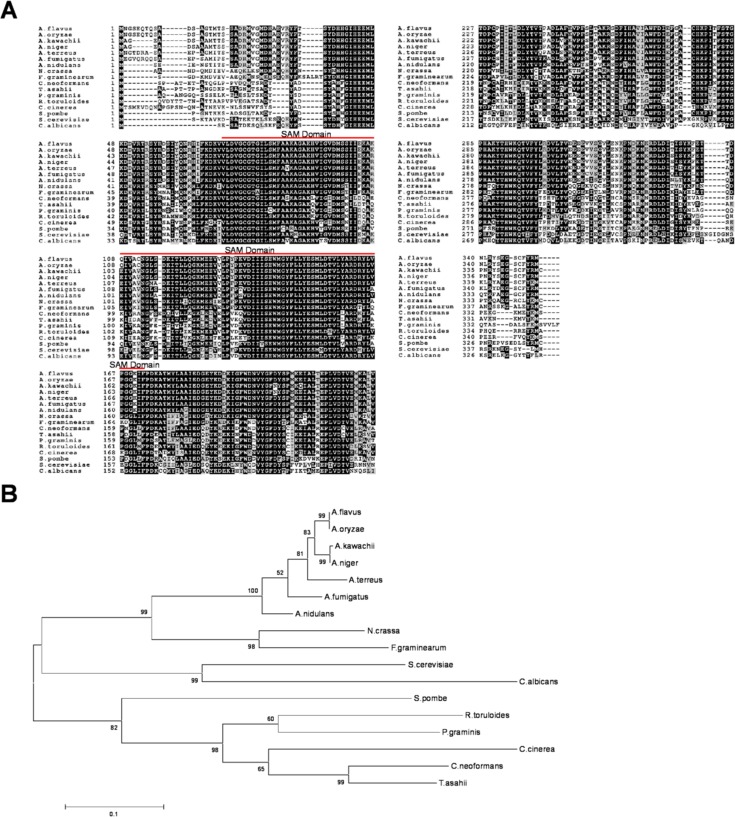
Multiple sequence alignment and phylogenetic analysis of RmtA and other fungal homologs. (A) Sequences aligned using Muscle (http://www.ebi.ac.uk/Tools/msa/muscle/). Alignment was visualized with BoxShade v3.21 (http://ch.embnet.org/software/BOX_form.html). (B) Phylogenetic tree of RmtA homologs from different fungal species. Phylogenetic trees constructed using MEGA v6.0. Trees were generated with Maximum-Likelihood model with a bootstrap value of 1000.

Comparative analysis of the *A*. *flavus* RmtA deduced amino acid sequence revealed significant identity (>50%) with putative homologs present in numerous fungal species (S2 Table). This trend was also identified among model eukaryotic species, with shared identity greater than 45% (S3 Table). Sequence alignment showed a highly conserved S-adenosyl methionine (SAM) binding domain among these putative homologs in other fungal species and other non-fungal eukaryotes (Fig 1A & S1A Fig). In addition, the RmtA phylogenetic tree was consistent with both fungal and other eukaryotes’ taxonomy (Fig 1B & S1B Fig) [39].

Comparison of *A*. *flavus* RmtA, RmtB, and RmtC deduced amino acid sequences revealed to be distinct from each other (S2 Fig), while they are highly conserved with respect to their corresponding homologs in *A*. *nidulans* (S2B Fig).

### *rmtA* Is Required for Normal Conidiation

**Fig 2:**
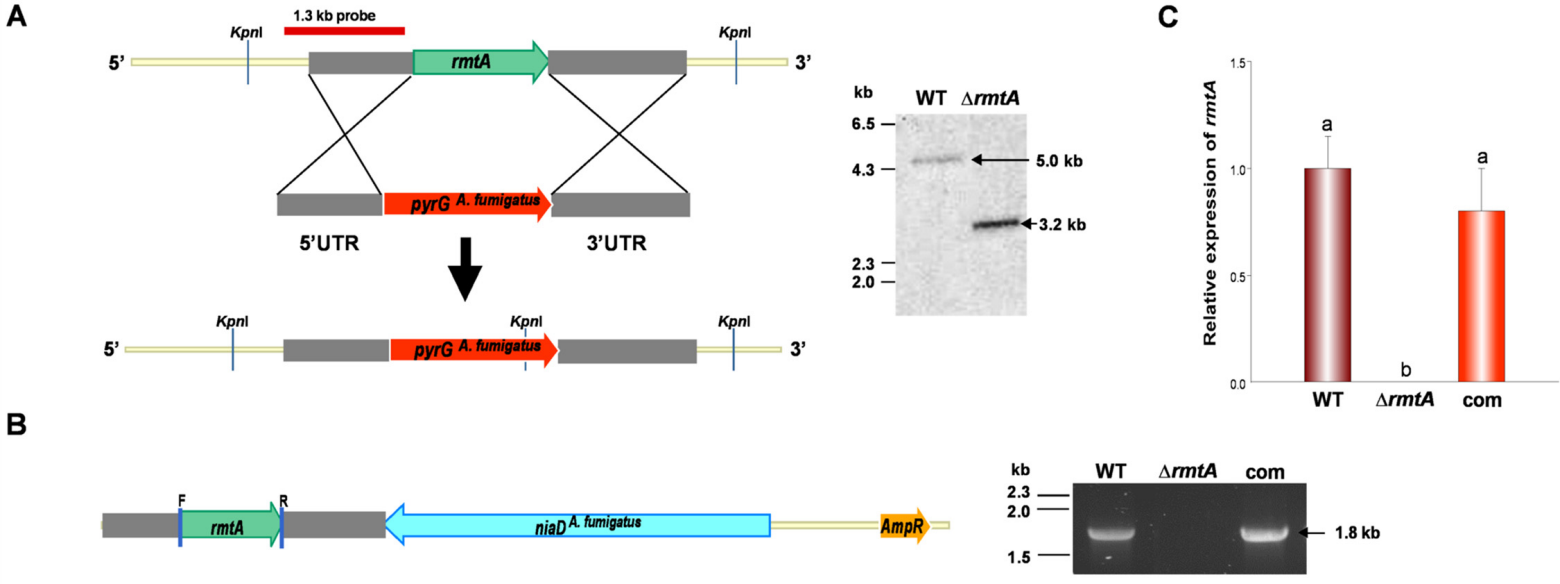
Construction of the *rmtA* deletion and complementation strains. (A) Diagram showing the gene replacement strategy using the selection marker *pyrG* from *A*. *fumigatus* and Southern blot analysis. Recombination events between the flanking regions are indicated with crosses. KpnI sites are indicated in both the wild-type and modified loci. A 1.3 kb fragment was used as probe as indicated. On the right, Southern blot image confirming of the deletion of *rmtA*. Genomic DNA from the wild type (WT) and from a selected deletion mutant Δ*rmtA* was digested with KpnI. Expected 5.0 kb and 3.2 kb bands are shown for WT and Δ*rmtA* respectively. (B) Linearized representation of the plasmid containing the *rmtA* wild-type allele. The *niaD* gene from *A*. *fumigatus* was used as selection marker for fungal transformation. Primers OE_RMTA_F and OE_RMTA_R (S1 Table), used for confirmation of the strain, are labeled in this figure as F and R respectively. On the right, gel electrophoresis results showing the presence of a 1.8 kb PCR product, confirming the presence of the wild-type allele in the complementation strain. Wild type and Δ*rmtA* were used as positive and negative control, respectively. (C) Expression analysis of *rmtA* by qRT-PCR. Strains were inoculated in PDB (10^6^ spores/ml), and cultures were grown for 48 h at 30°C. The error bars represent standard errors. Values were normalized to the expression levels in the wild type, considered as 1. Different letters on the columns indicate values that are statistically different (p < 0.05).

**Fig 3:**
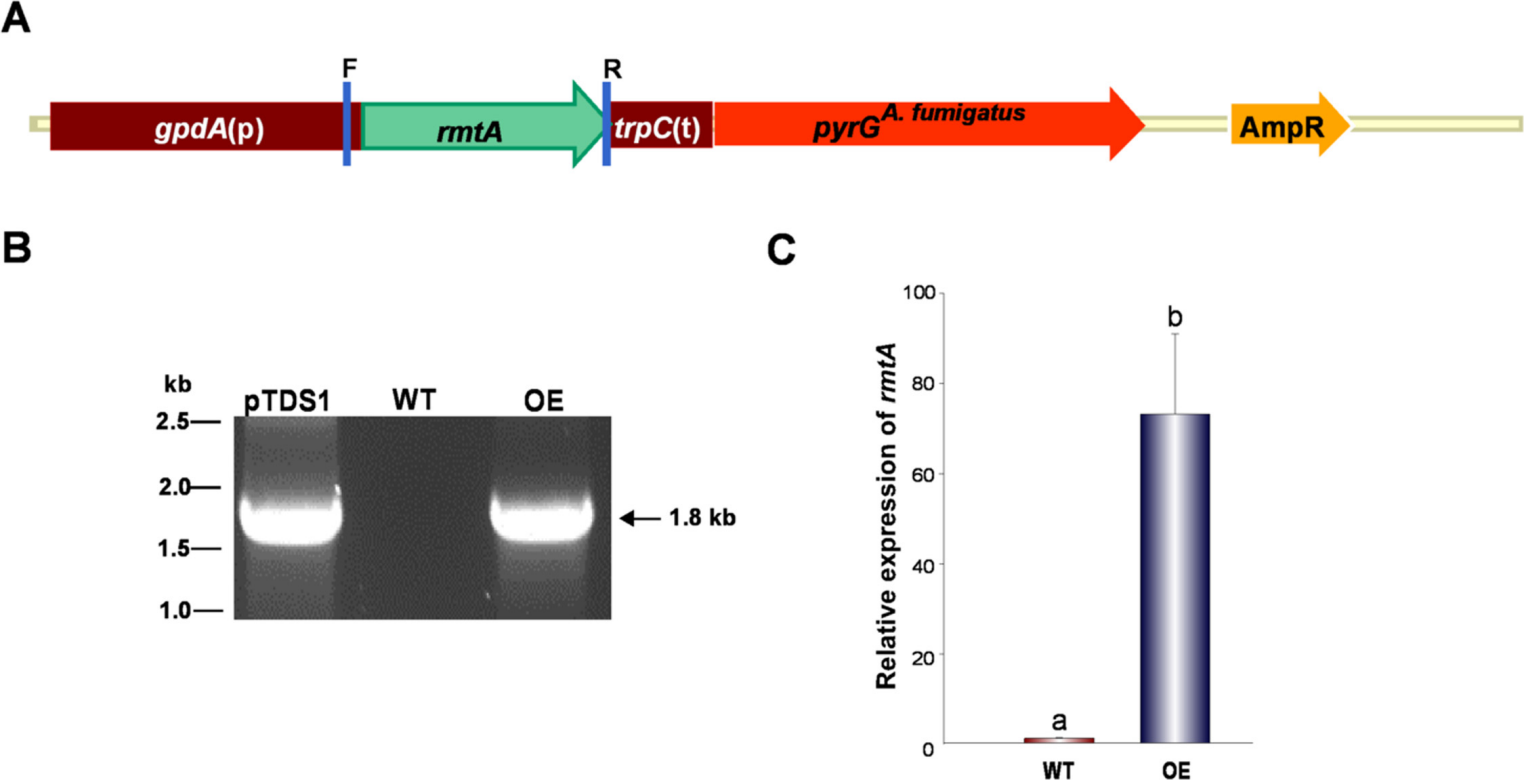
Confirmation of the OE*rmtA* (OE). (A) Linearized representation of the over-expression plasmid. The *pyrG* gene from *A*. *fumigatus* was used as a selection marker for fungal transformation. (B) Gel electrophoresis results showing the presence of a 1.8 kb PCR product, confirming the presence of the over-expression cassette. Plasmid pTRS.1rmtAOE and genomic DNA from the wild type were used as positive and negative control respectively. F and R represent primers OE_RMTA_F and OE_RMTA_R respectively. (C) Expression analysis of *rmtA* by qRT-PCR. Strains were inoculated in PDB (10^6^ spores/ml), and cultures were grown for 48 h at 30°C. The error bars represent standard errors. Values were normalized to the expression levels in the wild type, considered as 1. Different letters on the columns indicate values that are statistically different (p < 0.05).

To determine the effects of *rmtA* on morphogenesis and other cellular processes, *rmtA* deletion and complementation strains were constructed. The deletion strain was confirmed by Southern blot analysis (Fig 2A). Genomic DNA from both wild-type and Δ*rmtA* strains was isolated and digested with KpnI. A 1.3 kb DNA fragment corresponding to the 5’ UTR region of *rmtA* was used to generate the radioactive probe utilized in this hybridization. The presence of a 3.2 kb band in the Southern blot analysis confirmed the *rmtA* deletion (Fig 2A). With respect to the complementation strain, diagnostic PCR was used to verify the integration of the wild-type allele in the Δ*rmtA* strain (Fig 2B). qRT-PCR was utilized to confirm the lack of *rmtA* expression in the *rmtA* mutant under conditions that allow transcription of this gene in the wild-type and complementation control strain (Fig 2C). Additionally, an over-expression strain was created as described in the Materials and Methods section. Verification of this strain was carried out by diagnostic PCR (Fig 3), obtaining the expected 1.8 kb PCR product. Over-expression of *rmtA* was also confirmed by qRT-PCR (Fig 3C). All the strains generated in this study presented similar growth rate compared to the wild-type strain (S3 Fig).

**Fig 4:**
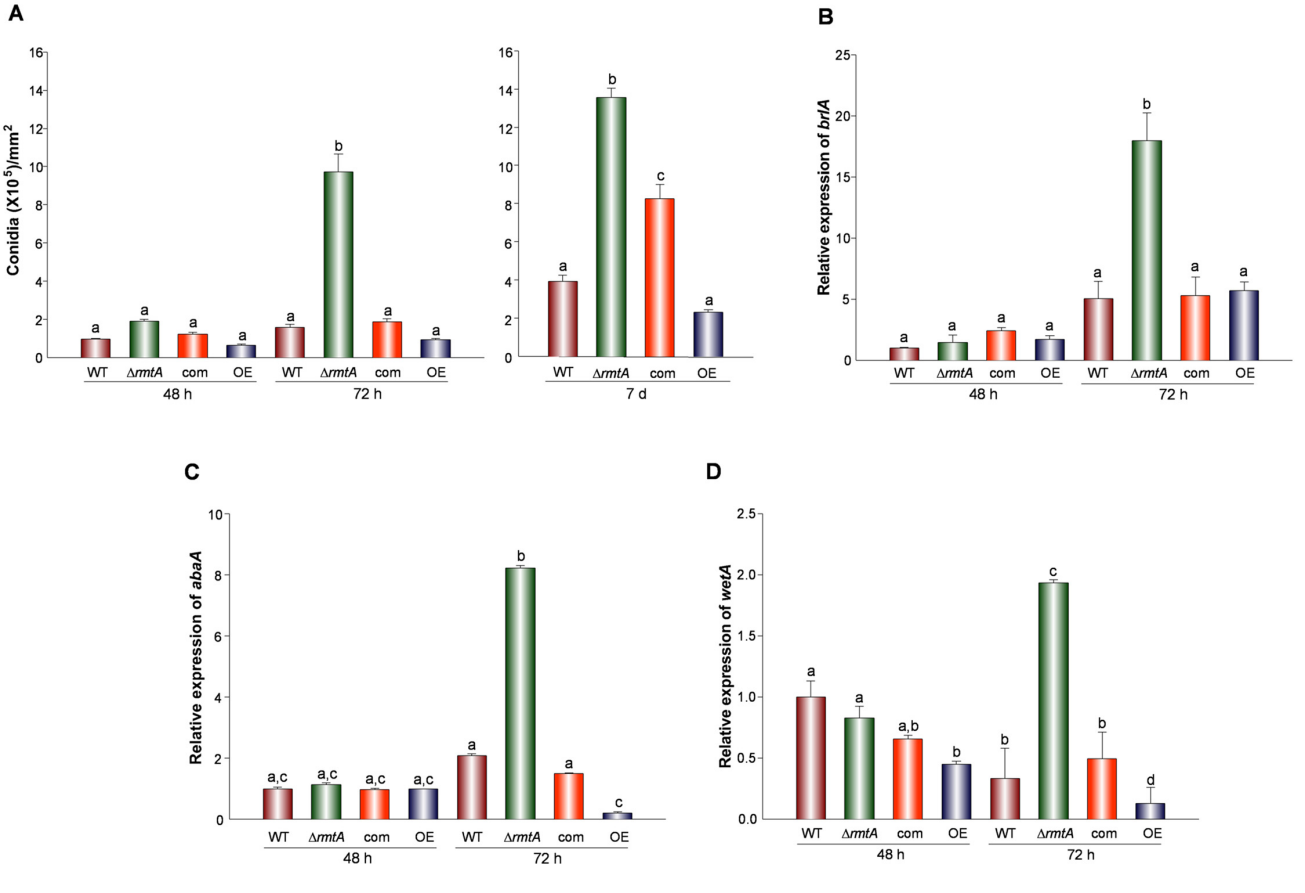
Effect of *rmtA* on conidiation. (A) Quantification of conidia. *Aspergillus flavus* wild type (WT), Δ*rmtA*, complementation (com) and OE*rmtA* (OE) strains were grown on PDA medium for up to 7 days at 30°C. Seven millimeter cores were taken from each culture. Conidia were counted using a hemocytometer. Values represent the average of 3 replicates. Error bars represent standard error. (B, C, D) qRT-PCR expression analysis of *brlA*. *abaA and wetA*, respectively. Strains were inoculated in PDB stationary cultures (10^6^ spores/ml), and were grown for 72 h at 30°C. The error bars represent standard errors. Values were normalized to the expression levels in the wild type, considered as 1. Different letters on the columns indicate values that are statistically different (p < 0.05).

Our study revealed a significant increase (5-fold) in conidial production in the Δ*rmtA* strain compared to the wild-type (Fig 4). Complementation with the wild-type allele rescued wild-type phenotype. While hypercondiation was observed in the Δ*rmtA*, over-expression of *rmtA* resulted in a reduction in conidial production (Fig 4A). Gene expression analysis showed that transcription of *brlA*, *abaA*, and *wetA*, genetic components of a key central regulatory pathway necessary for the activation of conidiation (reviewed by Krijgsheld et al, 2013[40]) is regulated by *rmtA*. Deletion of *rmtA* resulted in a significant increase in expression of all three genes (Fig 4B, 4C & 4D), while forced over-expression of *rmtA* led to a decrease in *abaA* and *wetA* expression levels.

### *rmtA* Affects Sclerotial Production

**Fig 5:**
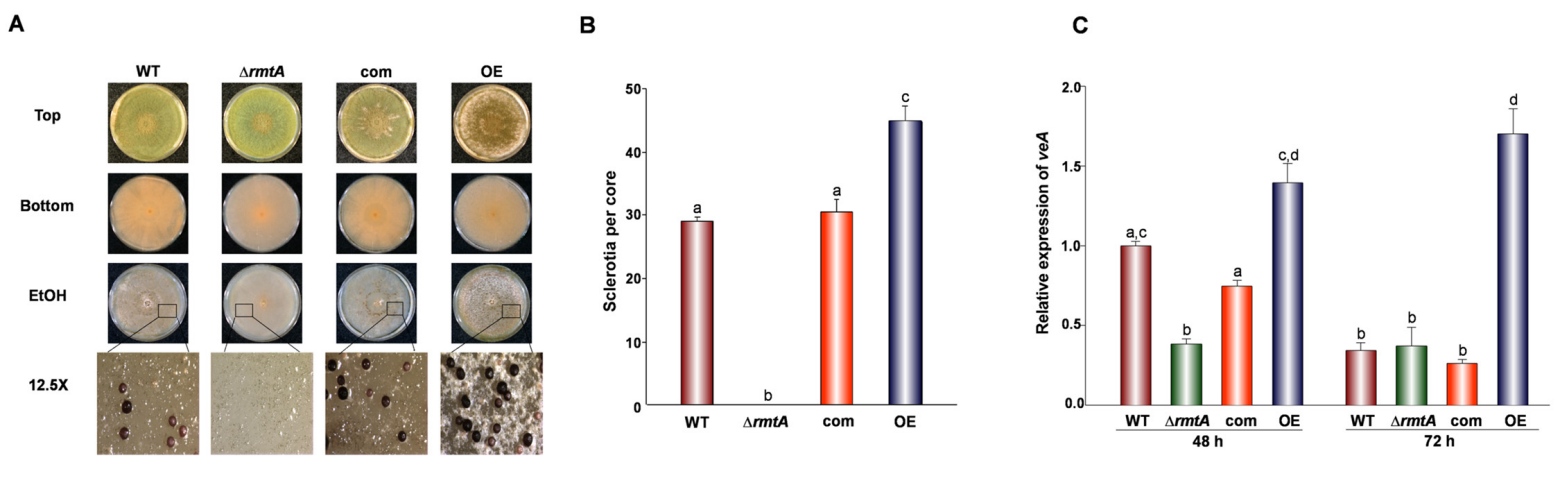
Effect of *rmtA* on sclerotial production on PDA. (A) *A*. *flavus* wild type (WT), Δ*rmtA*, complementation (com) and OE*rmtA* (OE) strains were point-inoculated on PDA medium and incubated for 9 days in the dark at 30°C. Photographs of cultures were taken before and after spraying 70% ethanol to remove conidia in order to improve visualization of sclerotia. Micrographs were obtained approximately 1.5 cm away from the center of the plate using a Leica MZ75 dissecting microscope at 12.5X magnification. (B) Quantification of sclerotia. *A*. *flavus* wild type, (WT), Δ*rmtA*, complementation (com) and OE *rmtA* strains grown on PDA medium for 24 days at 30°C. Sixteen millimeter cores were collected 1 cm away from the center. Number of sclerotia in each core were counted under a Leica MZ75 dissecting microscope. The experiment included 3 replicates. Error bars represent standard error. (C) qRT-PCR expression analysis of *veA*. *A*. *flavus* wild type (WT), Δ*rmtA*, complementation (com) and OE*rmtA* (OE) strains were inoculated in PDB stationary cultures (10^6^ spores/ml), and were grown for 48 and 72 h at 30°C. Error bars represent standard errors. Values were normalized to the expression levels in the wild type, considered as 1. Different letters on the columns indicate values that are statistically different (p < 0.05).

The Δ*rmtA* strain demonstrated a complete abolishment of sclerotial production when grown on PDA, a medium that allows production of these structures in the wild type (Fig 5). This pattern was observed even after 24 days of incubation. However, the over-expression presented a significant increase of sclerotia compared to the control strains. Similarly, on GMM with 2% sorbitol, another medium conducive to sclerotial production, the deletion *rmtA* strain did not produce any sclerotia (S4 Fig). Only on aged cultures growing on Wickerham medium, which is highly conducive to sclerotial production, a few sclerotia were produced by the deletion strain (S5 Fig). Interestingly, our study showed that *rmtA* positively influences the expression of the global regulator *veA* (Fig 5C), known to be necessary for sclerotial production in *A*. *flavus* (reviewed by Calvo and Cary, 2015 [2]). Our gene expression analysis revealed that at earlier time points *veA* transcription levels are reduced in Δ*rmtA* in comparison to wild-type. Furthermore, *veA* expression increases when expression of *rmtA* is abnormally increased in the OE*rmtA* strain.

### *rmtA* Is Required for Normal Aflatoxin B_1_ Biosynthesis and Production of Other Secondary Metabolites

**Fig 6:**
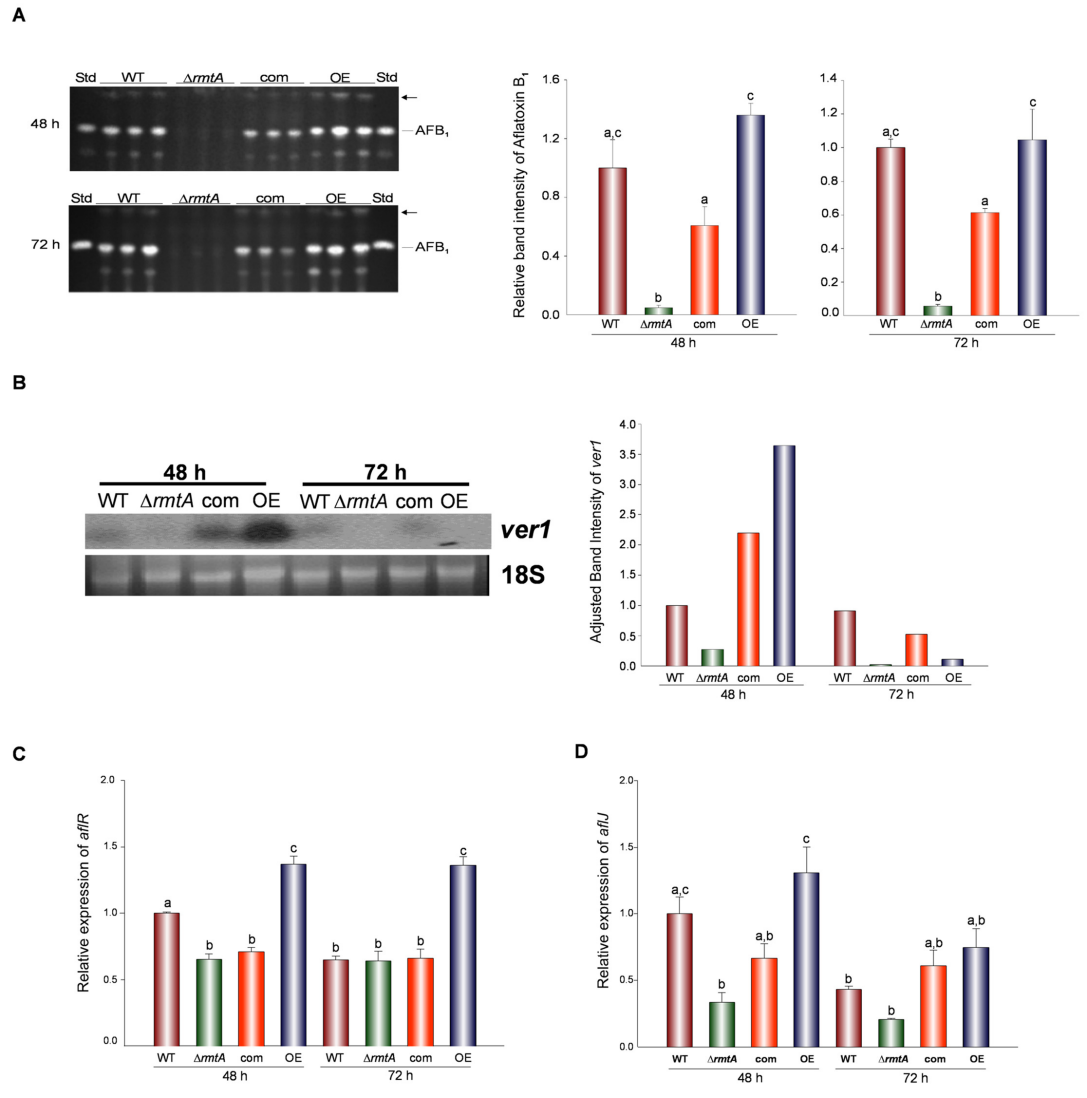
*rmtA* positively regulates Aflatoxin B_1_ production. (A) TLC analysis of aflatoxin B1 produced by *A*. *flavus* wild type (WT), Δ*rmtA*, complementation (com) and OE*rmtA* (OE) strains growing at 30°C on PDA top-agar inoculated cultures for 48 h and 72 h. Aflatoxin standard (AFB_1_) was also included on either side of the plate. Arrow indicates an unknown metabolite whose synthesis is also affected by *rmtA*. Densitometry of aflatoxin performed using GelQuantNET software is shown. (B) Northern Blot analysis of *ver1*. All strains were grown in PDB stationary cultures (10^6^ spores/ml) for 72 h at 30°C. 18S rRNA was used as loading control. Densitometry of Northern blot results is shown. (C & D) Expression analysis of *aflR* and *aflJ* by qRT-PCR respectively. The error bars represent standard errors. Different letters on the columns indicate values that are statistically different (p < 0.05).

Our TLC analysis revealed that Δ*rmtA* presented a decrease in aflatoxin B1 production compared to the wild-type strain at both time points analyzed (48 h and 72 h) (Fig 6). However, OE*rmtA* showed a slight increase in aflatoxin production with respect to the control strains. Additionally, our TLC analysis indicated that *rmtA* is necessary for the production of another metabolite (Fig 6A).

To determine the effect of *rmtA* on the expression of genes involved in aflatoxin biosynthesis, transcription levels of the structural gene *aflM*/*ver-1*, commonly used as indicator of aflatoxin cluster activation, as well as expression levels of the regulatory genes *aflR* and *aflS*/*aflJ* [41], were examined at 48 h and 72 h after inoculation (Fig 6B, 6C and 6D). Northern blot analysis of *aflM*/*ver-1* revealed that its expression was positively regulated by *rmtA*; while deletion of *rmtA* decreased expression of *aflM*/*ver-1*, over-expression of *rmtA* clearly enhanced expression of this structural gene at 48 h (Fig 6B). Our results also indicated that while deletion of *rmtA* did not affect *aflR*, an increase was observed in the over-expression strain (Fig 6C). Notably, deletion of *rmtA* resulted in a decrease in *aflS*/*aflJ* expression, while over-expression of *rmtA* increased it, particularly at 48 h (Fig 6D).

### Altered Expression of *rmtA* Affects Oxidative Stress Tolerance

**Fig 7:**
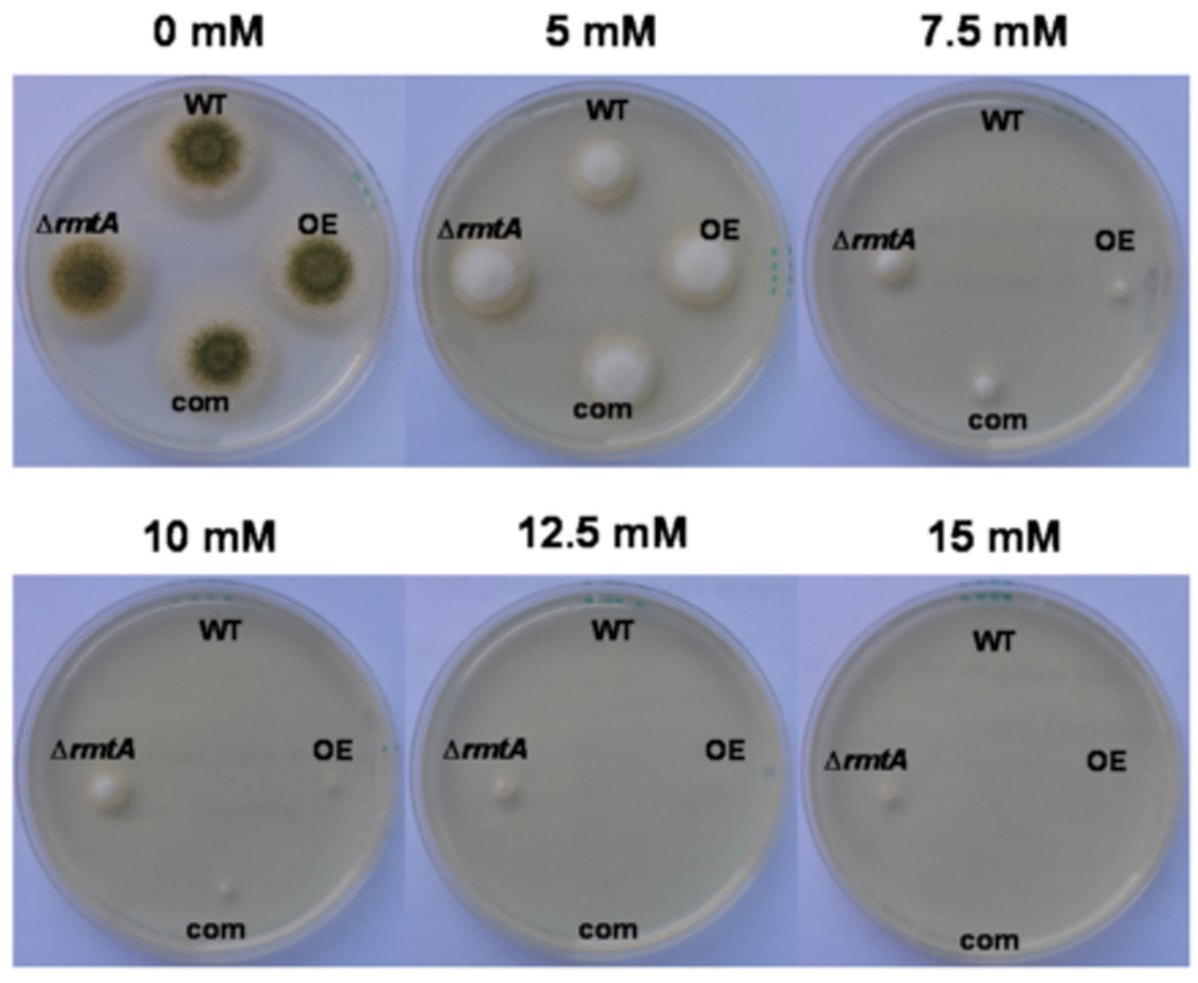
Role of *rmtA* in oxidative stress tolerance. *A*. *flavus* wild type (WT), Δ*rmtA*, complementation (com) and OE*rmtA* (OE) strains were point-inoculated on PDA containing a range of menadione concentrations (5 mM, 7.5 mM, 10 mM, 12.5 mM, and 15 mM). Cultures were incubated for 48 h at 30°C.

In order to evaluate the possible role of *rmtA* in oxidative stress response, *A*. *flavus* wild- type, Δ*rmtA*, complementation and OE*rmtA* strains were tested on medium containing different concentrations of menadione (5 mM to 15 mM). Under these conditions, Δ*rmtA* presented an increased tolerance to oxidative stress with respect to the other strains (Fig 7).

## Discussion

In this study we investigated the role of *rmtA*, encoding a putative type I arginine methyltransferase, in development and secondary metabolism of the agriculturally important fungus *A*. *flavus*. Our *in-silico* analysis revealed that the deduced protein, RmtA, is highly conserved within filamentous fungi, as well as in a diversity of eukaryotic organisms, including humans. As in the case of *A*. *flavus* RmtA, all RmtA homologs analyzed contain the SAM binding domain. Although the role of RmtA in histone methylation was demonstrated in the phylogenetically closely related fungus *A*. *nidulans* [29] the only phenotype reported in this study was an increase of oxidative stress sensitivity. The present study shows that in *A*. *flavus* several cellular processes are regulated by *rmtA*. Based our results, while the protein sequence is highly conserved, the regulatory output appears to vary depending on the species. Previous studies showed that *rmtA* is necessary for growth in *Neurospora crassa* [31], in *Fusarium graminearum* [30] and in the basidiomycete *Coprinopsis cinerea* [42]. However, our study showed that absence of *rmtA* does not affect growth in *A*. *flavus*. Additionally, the role of *rmtA* on oxidative stress tolerance in *A*. *flavus* differs from that described in *A*. *nidulans* and *F*. *graminearum*. While deletion of *rmtA* in both of these fungi results in a hypersensitivity to oxidative stress [29, 30], absence of *rmtA* in *A*. *flavus* increases resistance to this environmental condition. It is possible that the epigenetic mechanism involving *rmtA* in these fungal species diverged over the course of evolution resulting in variation in its regulatory scope leading to different adaptations suited for each species niche.

Several studies have associated other methyltransferases and other histone modifiers with regulation of secondary metabolism in fungi, including *Aspergillus* species. For example the histone acetyltransferase EsaA was shown to regulate production of sterigmatocystin, penicillin, terrequinone, and orsellinic acid in *A*. *nidulans* [43]. Another methyltransferase, LlmF, was also shown to be a negative regulator of sterigmatocystin in this model fungus [44]. In *A*. *flavus*, the putative methyltransferase LaeA is required for the production of cyclopiazonic acid, kojic acid, oryzaechlorin, aflatrem as well as aflatoxin [20]. Another example is the KMT6 histone methyltransferase in *Fusarium graminearum*, which has been reported to regulate both the fusarin C and carotenoid clusters [45]. Based on these reports, we examined whether *rmtA* was involved in the production of aflatoxin in *A*. *flavus*. Interestingly, our study revealed that *rmtA* is a positive regulator of aflatoxin biosynthesis and associated aflatoxin cluster genes. Our experiments showed that expression of *aflJ* (also termed *aflS* [41]) was positively regulated by *rmtA*. *aflJ* is a regulatory gene in the aflatoxin gene cluster that encodes a protein that promotes the activation of early genes in this cluster [41]. AflJ protein has been demonstrated to interact with AflR, a well-characterized transcription factor necessary for aflatoxin cluster activation [41]. Over-expression of *rmtA* resulted in higher expression of not only *aflJ* but also of *aflR*.

Interestingly, the synthesis of another metabolite was also affected by the absence of *rmtA*. This suggests a broader regulatory scope of *rmtA* on secondary metabolism in *A*. *flavus*. A study of the *rmtA* homolog, *amt1*, in *Fusarium graminearum* showed this gene as a positive regulator of the synthesis of deoxynivalenol, a harmful compound that is produced during *Fusarium* head blight [30]. It is likely that this aspect of *rmtA* regulation, involving the control of biosynthesis of natural products, might be conserved in other fungal species.

In fungi secondary metabolism is genetically linked to morphological development [14–16, 46]. Our study indicates that *rmtA* not only controls secondary metabolism, but it is also a regulator of conidiation in *A*. *flavus*. Air-borne conidiospores, or conidia, are an efficient way of fungal dissemination [47], which is particularly relevant in *A*. *flavus* field infestations. In addition, *A*. *flavus* can cause aspergillosis, particularly in immunocompromised patients, where conidia constitute the main inoculum [48]. In our study, absence of *rmtA* resulted in hyperconidiating colonies. This increase in the production of conidia coincided with an increase in the expression of *brlA*, *abaA*, and *wetA*. In *Aspergillus*, *brlA* is a regulatory gene that encodes a C_2_H_2_ zinc finger transcription factor [49] that initiates a central regulatory pathway that governs maturation of conidiophores vesicles. BrlA activates other regulatory genes such as *abaA*, which acts as a transcriptional switch controlling *wetA*, which activates the expression of spore-specific gene [40, 49]. This, together with the results of our study, suggests that the developmental effect of *rmtA* on conidiation is, at least in part, mediated by *brlA*. In addition, abnormally high *rmtA* transcription levels in the over-expression strain resulted in a slight reduction in conidiation which can be explained by lower expression levels of *abaA* and *wetA*. This result further supports the role of *rmtA* as negative regulator of asexual development in *A*. *flavus*.

Besides the effect of *rmtA* on conidiation, *rmtA* also affected sclerotial production. Sclerotia are structures composed of a matrix of hardened mycelia that allow *A*. *flavus* to survive adverse environmental conditions [50]. They are vestiges of fruiting bodies that in most cases lost the capacity to produce sexual spores; although formation of ascospores within sclerotia, termed stromata, has been observed under laboratory conditions [51]. When conditions are favorable again sclerotia will produce hyphae to establish a new mycelium and/or generates conidiophores on their surface further contributing to disseminate the fungus [52]. Our study showed that deletion of *rmtA* strongly reduces or blocks sclerotial production, while over-expression of *rmtA* resulted in hyper-production of these resistant structures, indicating that *rmtA* is a positive regulator of sclerotial formation. Interestingly, our study show that *rmtA* positively affects the expression of *veA*, encoding a global regulator that forms part of the nuclear *velvet* protein complex [18]. *veA* has been shown to be essential for sclerotial production in *A*. *flavus* [19]. Based on our results it is likely that the effect of *rmtA* on sclerotia could be affected by *veA*. In addition, *veA* has been shown to regulate other cellular processes in fungi, including conidiation and secondary metabolism [2, 53–60]. Therefore, other roles of *rmtA* described in this study could also be *veA*-dependent, for example *veA* is a repressor of conidiation [58, 60], and it is possible that the observed negative effect of *rmtA* on conidiation, as well as its positive effect on secondary metabolism, could also be influenced by the effect of *rmtA* on *veA* expression in *A*. *flavus*. As in our study, functional association of VeA with other methyltransferases, such as LlmF, VipC, VapB, and LaeA, that affect development and secondary metabolism has been previously described [18, 44, 61, 62].

In conclusion, this study contributes to the elucidation of *rmtA* functions in *A*. *flavus*, revealing high conservation among its homologs in many eukaryotes. Despite this conservation, the regulatory role of *rmtA* varies among fungal species, suggesting that “rewiring” of this regulatory mechanism has occurred through evolution. This study also shows that morphogenesis is under *rmtA* regulation, influencing the developmental balance between conidiation and sclerotial formation in *A*. *flavus*; *rmtA* negatively regulates conidial production while it promotes sclerotial development. In addition, we also demonstrated that *rmtA* positively regulates secondary metabolism, controlling the production of aflatoxin as well as the synthesis of another unknown metabolite. Other cellular processes are also under *rmtA* regulation, including oxidative stress response. These facts indicate that *rmtA* is a global regulator in *A*. *flavus*. Furthermore, we found that *rmtA* control the expression of the master regulator *veA*, suggesting that *rmtA* regulatory output is functionally connected with *veA*. The findings in this study could contribute to set the bases of novel control strategies to reduce the negative impact caused by *A*. *flavus* and other detrimental fungal species.

## Supporting Information

S1 Fig Multiple sequence alignment and phylogenetic analysis of RmtA and other homologs in model organisms. (A) Sequences aligned using Muscle (http://www.ebi.ac.uk/Tools/msa/muscle/). Alignment was visualized with BoxShade v3.21 (http://ch.embnet.org/software/BOX_form.html). (B) Phylogenetic tree of RmtA homologs from model organisms. Phylogenetic trees constructed using MEGA v6.0. Trees were generated with Maximum-Likelihood model with a bootstrap value of 1000.
(PDF)

S2 Fig Multiple sequence alignment of *A*. *flavus* RmtA, RmtB, and RmtC, and phylogenetic analysis of these protein sequences and those of their putative homologs in *A*. *nidulans*. (A) Sequences alignment of *A*. *flavus* RmtA, RmtB (EED57275.1), and RmtC (EED50528.1) using Muscle (http://www.ebi.ac.uk/Tools/msa/muscle/). Alignment was visualized with BoxShade v3.21 (http://ch.embnet.org/software/BOX_form.html). (B) Phylogenetic tree of RmtA, RmtB, and RmtC from *A*. *flavus* and *A*. *nidulans*. Accession numbers for *A*. *nidulans* RmtB and RmtC sequence are XP_660700.1 and XP_657738.1 respectively. The phylogenetic tree was constructed using MEGA v6.0, and it was generated with Maximum-Likelihood model with a bootstrap value of 1000.
(PDF)

S3 Fig Evaluation of colony growth. (A) Images of point-inoculated cultures of *A*. *flavus* wild type (WT), Δ*rmtA*, complementation (com) and OE*rmtA* (OE) strains growing on PDA after 7 days of incubation at 30°C. (B) Quantification of colony growth as colony diameter of cultures in (A). Different letters on the columns indicate values that are statistically different (p < 0.05).
(PDF)

S4 Fig Effect of *rmtA* on sclerotial production on GMM-sorbitol. *A*. *flavus* wild type (WT), Δ*rmtA*, complementation (com) and OE*rmtA* (OE) strains were point-inoculated and grown on GMM-sorbitol medium for 7 days at 30°C. Plates were then sprayed with ETOH and micrographs were taken approximately 1.5 cm from center at 12.5X magnification using a Leica MZ75 dissecting microscope coupled with a Leica DC SOLP camera.
(PDF)

S5 Fig Effect of *rmtA* on sclerotial production on Wickerham medium. *A*. *flavus* wild type (WT), Δ*rmtA*, complementation (com) and OE*rmtA* (OE) strains were point-inoculated on Wickerham medium and incubated for 7 days (A) and 17 days (B) at 30°C. Plates were then sprayed with ethanol and micrographs of sclerotia were obtained approximately 1.5 cm from the center at 12.5X magnification using a Leica MZ75 dissecting microscope.
(PDF)

S1 Table Primers used in this study. (PDF)

S2 Table Sequence comparison of RmtA in other fungal species. (PDF)

S3 Table Sequence Comparison of RmtA in eukaryotic model organisms. (PDF)
