# Supplementary material for: RmtA, a Putative Arginine Methyltransferase, Regulates Secondary Metabolism and Development in Aspergillus flavus
Source: PLoS One. 2016 May 23;11(5):e0155575. doi: 10.1371/journal.pone.0155575 (PMC4877107; doi:10.1371/journal.pone.0155575)

S1 Fig. Multiple sequence alignment and phylogenetic analysis of RmtA and other homologs in model organisms. (A) Sequences aligned using Muscle (http://www.ebi.ac.uk/Tools/msa/muscle/). Alignment was visualized with BoxShade v3.21 (http://ch.embnet.org/software/BOX_form.html). (B) Phylogenetic tree of RmtA homologs from model organisms. Phylogenetic trees constructed using MEGA v6.0. Trees were generated with Maximum-Likelihood model with a bootstrap value of 1000.

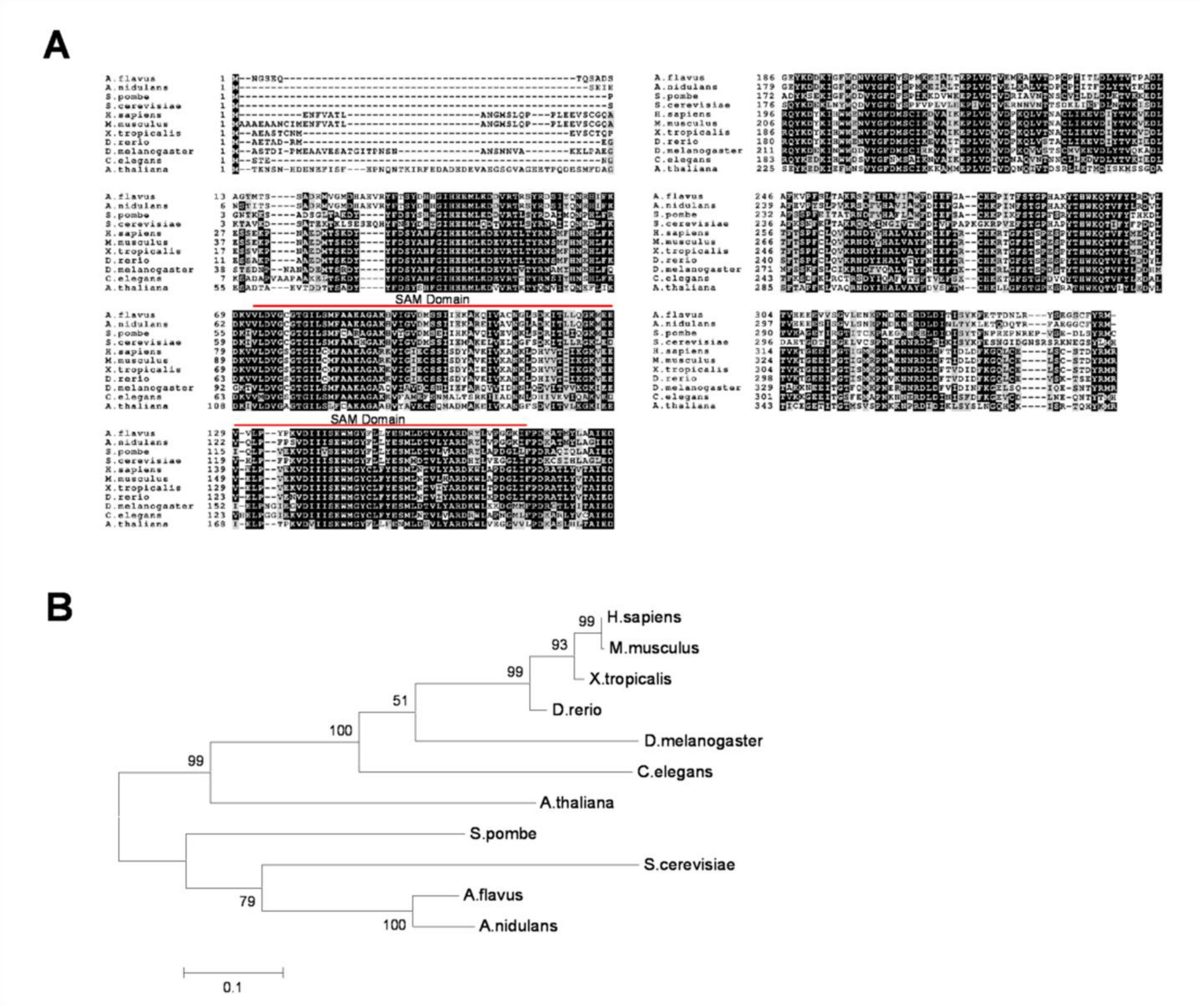

Supplement: S1 Fig — (A) Sequences aligned using Muscle (http://www.ebi.ac.uk/Tools/msa/muscle/). Alignment was visualized with BoxShade v3.21 (http://ch.embnet.org/software/BOX_form.html). (B) Phylogenetic tree of RmtA homologs from model organisms. Phylogenetic trees constructed using MEGA v6.0. Trees were generated with Maximum-Likelihood model with a bootstrap value of 1000. (PDF) [file pone.0155575.s001.pdf]
