# Supplementary material for: RmtA, a Putative Arginine Methyltransferase, Regulates Secondary Metabolism and Development in Aspergillus flavus
Source: PLoS One. 2016 May 23;11(5):e0155575. doi: 10.1371/journal.pone.0155575 (PMC4877107; doi:10.1371/journal.pone.0155575)

S2 Fig. Multiple sequence alignment of *A. flavus* RmtA, RmtB, and RmtC, and phylogenetic analysis of these protein sequences and those of their putative homologs in *A. nidulans*. (A) Sequences alignment of *A. flavus* RmtA, RmtB (EED57275.1), and RmtC (EED50528.1) using Muscle (http://www.ebi.ac.uk/Tools/msa/muscle/). Alignment was visualized with BoxShade v3.21 (http://ch.embnet.org/software/BOX_form.html). (B) Phylogenetic tree of RmtA, RmtB, and RmtC from *A. flavus* and *A. nidulans*. Accession numbers for *A. nidulans* RmtB and RmtC sequence are XP_660700.1 and XP_657738.1 respectively. The phylogenetic tree was constructed using MEGA v6.0, and it was generated with Maximum-Likelihood model with a bootstrap value of 1000.

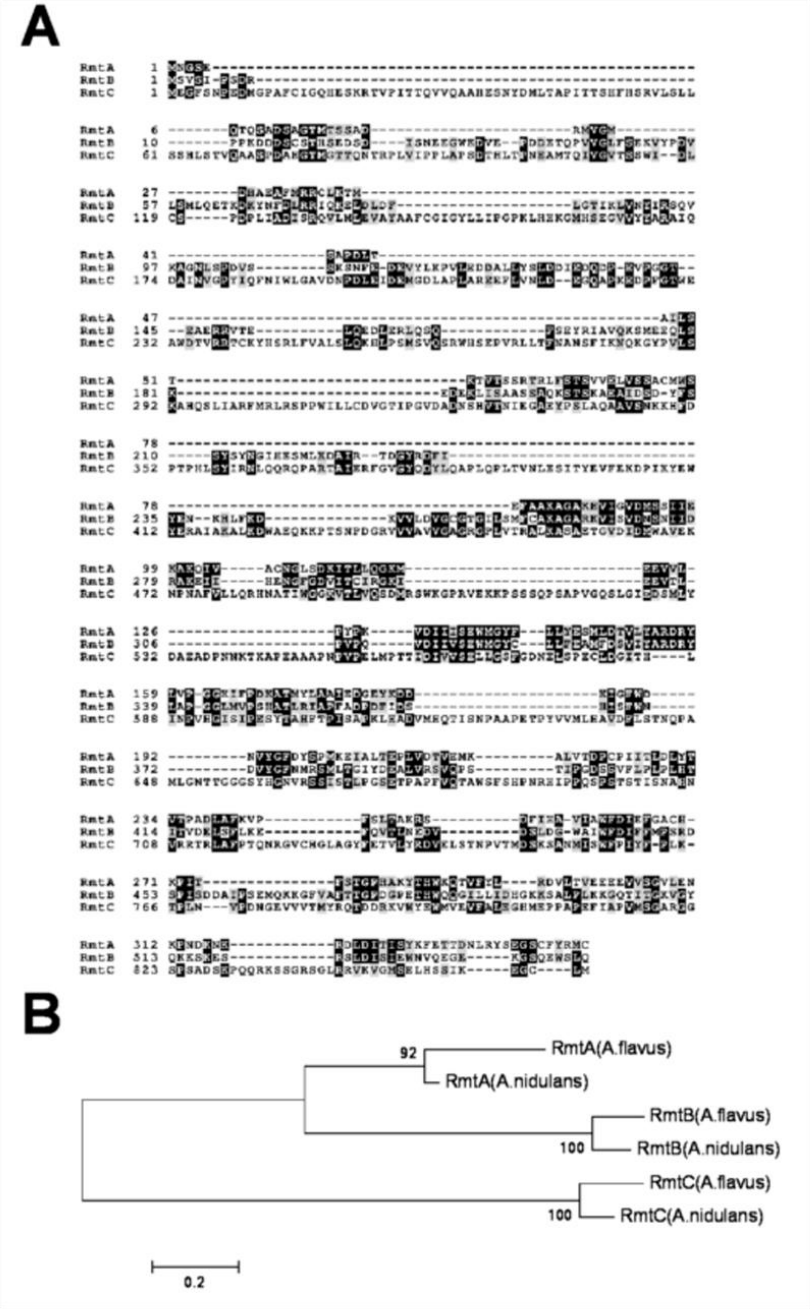

Supplement: S2 Fig — (A) Sequences alignment of A. flavus RmtA, RmtB (EED57275.1), and RmtC (EED50528.1) using Muscle (http://www.ebi.ac.uk/Tools/msa/muscle/). Alignment was visualized with BoxShade v3.21 (http://ch.embnet.org/software/BOX_form.html). (B) Phylogenetic tree of RmtA, RmtB, and RmtC from A. flavus and A. nidulans. Accession numbers for A. nidulans RmtB and RmtC sequence are XP_660700.1 and XP_657738.1 respectively. The phylogenetic tree was constructed using MEGA v6.0, and it was generated with Maximum-Likelihood model with a bootstrap value of 1000. (PDF) [file pone.0155575.s002.pdf]
