# Supplementary material for: RmtA, a Putative Arginine Methyltransferase, Regulates Secondary Metabolism and Development in Aspergillus flavus
Source: PLoS One. 2016 May 23;11(5):e0155575. doi: 10.1371/journal.pone.0155575 (PMC4877107; doi:10.1371/journal.pone.0155575)

S3 Fig. Evaluation of colony growth. (A) Images of point-inoculated cultures of *A. flavus* wild type (WT), Δ*rmtA,* complementation (com) and OE*rmtA* (OE) strains growing on PDA after 7 days of incubation at 30 °C. (B) Quantification of colony growth as colony diameter of cultures in (A). Different letters on the columns indicate values that are statistically different ($p < 0.05$).

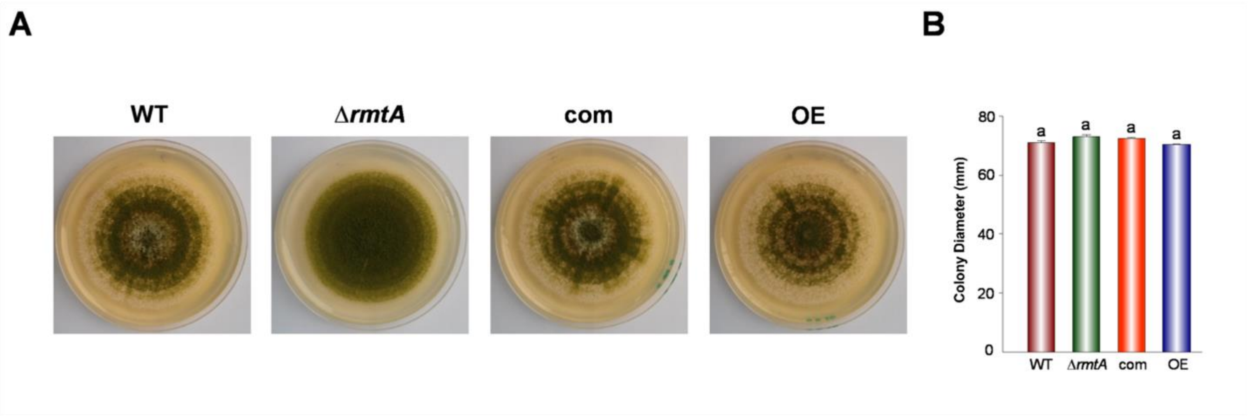

Supplement: S3 Fig — (A) Images of point-inoculated cultures of A. flavus wild type (WT), ΔrmtA, complementation (com) and OErmtA (OE) strains growing on PDA after 7 days of incubation at 30°C. (B) Quantification of colony growth as colony diameter of cultures in (A). Different letters on the columns indicate values that are statistically different (p < 0.05). (PDF) [file pone.0155575.s003.pdf]
