# Supplementary material for: RmtA, a Putative Arginine Methyltransferase, Regulates Secondary Metabolism and Development in Aspergillus flavus
Source: PLoS One. 2016 May 23;11(5):e0155575. doi: 10.1371/journal.pone.0155575 (PMC4877107; doi:10.1371/journal.pone.0155575)

S4 Fig. Effect of *rmtA* on sclerotial production on GMM-sorbitol. *A. flavus* wild type (WT), Δ*rmtA,* complementation (com) and OE*rmtA* (OE) strains were point-inoculated and grown on GMM-sorbitol medium for 7 days at 30 ºC. Plates were then sprayed with ETOH and micrographs were taken approximately 1.5 cm from center at 12.5X magnification using a Leica MZ75 dissecting microscope coupled with a Leica DC SOLP camera.

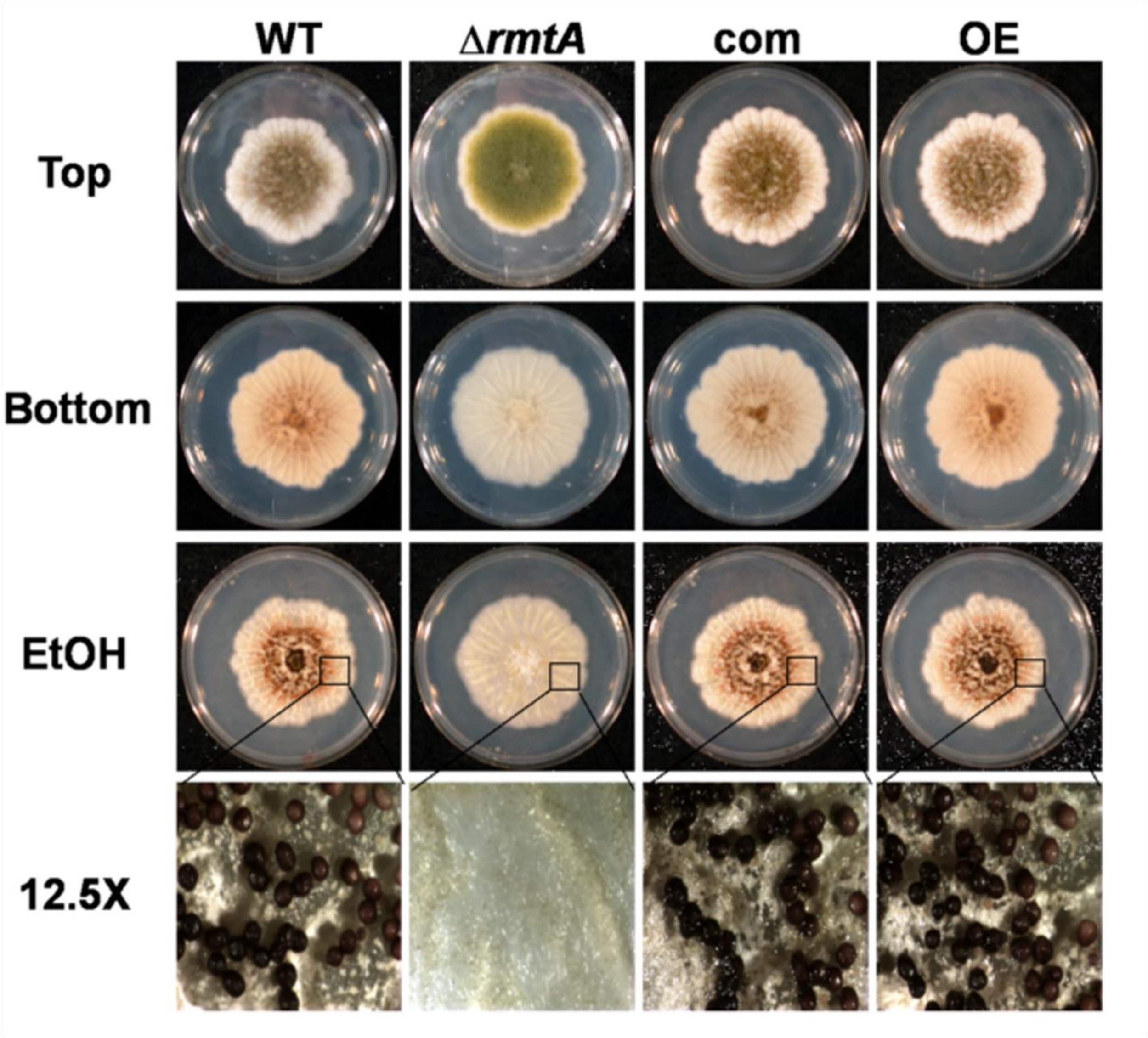

Supplement: S4 Fig — A. flavus wild type (WT), ΔrmtA, complementation (com) and OErmtA (OE) strains were point-inoculated and grown on GMM-sorbitol medium for 7 days at 30°C. Plates were then sprayed with ETOH and micrographs were taken approximately 1.5 cm from center at 12.5X magnification using a Leica MZ75 dissecting microscope coupled with a Leica DC SOLP camera. (PDF) [file pone.0155575.s004.pdf]
