# Supplementary material for: RmtA, a Putative Arginine Methyltransferase, Regulates Secondary Metabolism and Development in Aspergillus flavus
Source: PLoS One. 2016 May 23;11(5):e0155575. doi: 10.1371/journal.pone.0155575 (PMC4877107; doi:10.1371/journal.pone.0155575)

S5 Fig. Effect of *rmtA* on sclerotial production on Wickerham medium. *A. flavus* wild type (WT), Δ*rmtA,* complementation (com) and OE*rmtA* (OE) strains were point-inoculated on Wickerham medium and incubated for 7 days (A) and 17 days (B) at 30 ºC. Plates were then sprayed with ethanol and micrographs of sclerotia were obtained approximately 1.5 cm from the center at 12.5X magnification using a Leica MZ75 dissecting microscope.

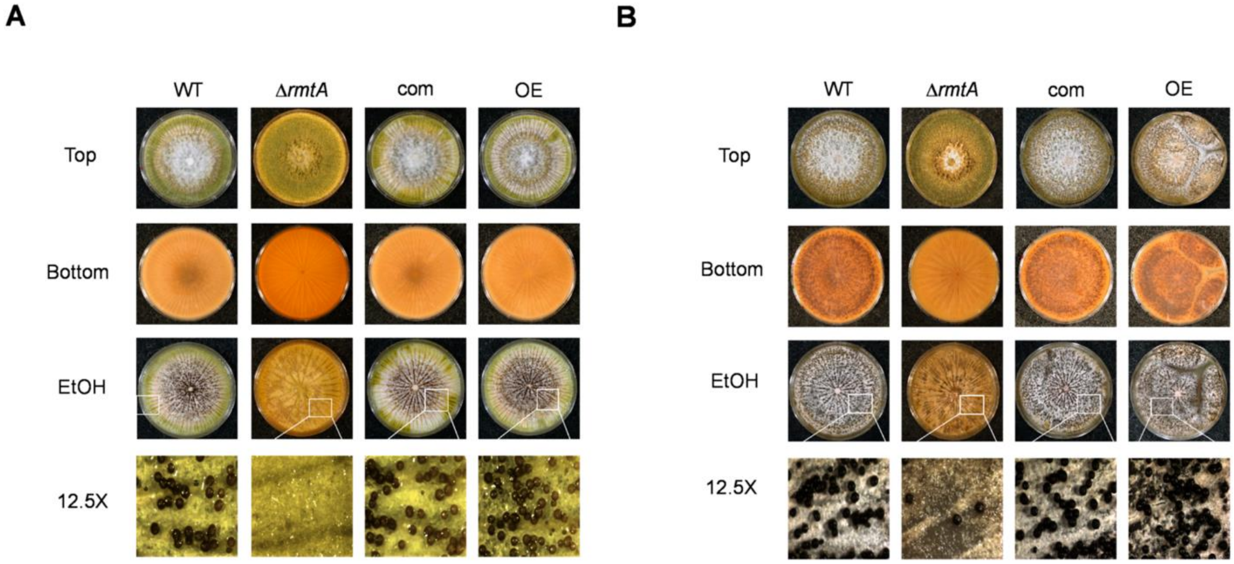

Supplement: S5 Fig — A. flavus wild type (WT), ΔrmtA, complementation (com) and OErmtA (OE) strains were point-inoculated on Wickerham medium and incubated for 7 days (A) and 17 days (B) at 30°C. Plates were then sprayed with ethanol and micrographs of sclerotia were obtained approximately 1.5 cm from the center at 12.5X magnification using a Leica MZ75 dissecting microscope. (PDF) [file pone.0155575.s005.pdf]
