# Supplementary material for: RmtA, a Putative Arginine Methyltransferase, Regulates Secondary Metabolism and Development in Aspergillus flavus
Source: PLoS One. 2016 May 23;11(5):e0155575. doi: 10.1371/journal.pone.0155575 (PMC4877107; doi:10.1371/journal.pone.0155575)
Supplement: S1 Table — (PDF) [file pone.0155575.s006.pdf]

Table S1. Primers used in this study.

Name	Sequence (5'→3')
Afl_rmtA_p1	CGAGATAAGCCGTGCCAATGGGC
Afl_rmtA_p2	GAAAGAAAAGAAGCAAGCAAACTAGAGGG
Afl_rmtA_p3	GTATTGGATCTGGTGAATCGTGGCACG
Afl_rmtA_p4	CACAGGCGAAGCAAAGCGTGCCT
Afl_rmtA_p5	CCCTCTAGTTTGCTTGCTTCTTTTCTTTCACCGGTCGCCTCAAACAATGCTCT
Afl_rmtA_p6	CGTGCCACGATTCACCAGATCCAATACGTCTGAGAGGAGGCACTGATGCG
Afl_rmtA_p7	CGGAGATGGAAAGCTAGGGAGGACG
Afl_rmtA_p8	CGAGGTGGAGACTATCTCACCGATAGAG
AFL_ RMTA_F	ATAGAGGGATCCGGATGAACGGATCGGAGCAGACACAATCCATAGAGGGATCC GGATGAACGGATCGGAGCAGACACAATCC
AFL_RMTA_R	TAGTGCTCGAGTTACATTCTATAGAAGCAGCTACCCTCGGAGTAACG
12228-pyrG_Afum_F	GATGTGACGACAACCCGAGAACTCC
12229-pyrG_Afum_R	GAGCAGCGTAGATGCCTCGAC
comp RMTA_flavus_F	NNNNNNNCTGCAGGGAATATCCTGATAATTGCCAGG
comp RMTA_flavus_R	NNNNNNNNNNNGCGGCCGCCCAATAACCGTATTACGCAACT
OE_RMTA_F	NNNNNNGGCGCGCCATGAACGGATCGGAGCAGACAC
OE_RMTA_R	NNNNNNGCGGCCGCCGGGGTATCTACTTGTGTAATTCGAGGAGC
gdpApromoF	AAGTACTTTGCTACATCCATACTCC
ver1-Nor-S	ATGTCCGACAACCACCGTTTAG
ver1-Nor-A	TCATCGAAAAGCGCCGCCA
qPCR-Afla rmta_F	CTTCTCTCTTACCGCCAAGCGCAGCGACTTCATCC
qPCR-Afla rmta_R	GGGCTTGTTCTCCAACACTCCCGAAACGACTTCCTC
qPCR-Afla_18S_F	TGATGACCCGCTCGGCACCTTACGAGAAATCAAAGT
qPCR-Afla_18S_R	GGCCATGCACCACCATCCAAAAGATCAAGAAAGAGC
qPCR-Afla_brlA_F	TATCCAGACATTCAAGACGCACAG
qPCR-Afla_brlA_R	GATAATAGAGGGCAAGTTCTCCAAAG
qPCR-Afla_aflR_F	GCAACCTGATGACGACTGATATGG
qPCR-Afla_aflR_R	TGCCAGCACCTTGAGAACGATAAG
qPCR-Afla_aflJ_F	TGGAATATGGCTGTAGGAAGTG
qPCR-Afla_aflJ_R	CATCCGAGTGAGCGTATCC
qPCR_Afla_abaA_F	GTACGGGAGCAACCAGCAGAC
qPCR_Afla_abaA_R	GTTGCCTCGGCGTGCAAATT
qPCR_Afla_wetA_F	GGGCTGTTCACGCCTGATCT
qPCR_Afla_wetA_R	GACCCCCTTGCAGGATGTCA
qPCR_Afla_veA_F	ATCATCCAGCAGTACCTCTCCAGAGCCCCAGTCACGA
qPCR_Afla_veA_R	CCGACTGGCATCTGACAGCCTCCGAGGATAACTTTC
