# Supplementary material for: RmtA, a Putative Arginine Methyltransferase, Regulates Secondary Metabolism and Development in Aspergillus flavus
Source: PLoS One. 2016 May 23;11(5):e0155575. doi: 10.1371/journal.pone.0155575 (PMC4877107; doi:10.1371/journal.pone.0155575)
Supplement: S2 Table — (PDF) [file pone.0155575.s007.pdf]

S2 Table. Sequence comparison of RmtA in other fungal species.

Species	Accession #	E-Value	Similarity	Identity
Aspergillus oryzae	XP_001818952.2	0	100	100
Aspergillus terreus	XP_001212165.1	0	96	91.5
Aspergillus fumigatus	XP_750368.2	0	93.5	89.5
Aspergillus kawachii	GAA89995.1	0	94.9	93.5
Aspergillus niger	XP_001398973.2	0	95.2	94.1
Aspergillus nidulans	CBF74424.1	0	91.2	86.9
Neurospora crassa	XP_963910.1	0	82.7	71.1
Fusarium graminearum	ESU06416.1	0	80.4	69
Cryptococcus neoformans	XP_569187.1	8.00E-153	73.7	59.6
Schizosaccharomyces pombe	NP_594825.2	9.00E-153	73	62.3
Saccharomyces cerevisiae	NP_009590.1	1.00E-148	71.2	59.2
Candida albicans	XP_718383.1	6.00E-133	68.2	53.8
Rhodosporidium toruloides	EMS18785.1	3.00E-158	75.9	61.9
Puccinia graminis	XP_003335266.2	2.00E-156	73.6	59.7
Trichosporon asahii	EJT52753.1	3.00E-155	73.8	61.3
Coprinopsis cinerea	XP_001830750.2	6.00E-143	69.4	56.7
