# Supplementary material for: RmtA, a Putative Arginine Methyltransferase, Regulates Secondary Metabolism and Development in Aspergillus flavus
Source: PLoS One. 2016 May 23;11(5):e0155575. doi: 10.1371/journal.pone.0155575 (PMC4877107; doi:10.1371/journal.pone.0155575)
Supplement: S3 Table — (PDF) [file pone.0155575.s008.pdf]

S3 Table. Sequence Comparison of RmtA in eukaryotic model organisms.

Species	Accession #	E-Value	Similarity	Identity
Aspergillus nidulans	CBF74424.1	0	91.2	86.9
Schizosaccharomyces pombe	NP_594825.2	9.00E-153	73	62.3
Saccharomyces cerevisiae	NP_009590.1	1.00E-148	71.2	59.2
Homo sapiens	CAA71763.1	9.00E-136	70.4	55.2
Arabidopsis thaliana	NP_194680.1	3.00E-135	62.1	47.2
Xenopus (Silurana) tropicalis	NP_001005629.2	3.00E-135	69.4	54.3
Danio rerio	XP_005164044	9.00E-135	68.1	54.4
Mus musculus	NP_062804	1.00E-134	66.2	51.7
Drosophila melanogaster	CAL26190.1	1.00E-133	66	49.7
Caernorhabditis elegans	NP_507909.1	2.00E-125	67.9	52.5
